# The transition of reported pain in different body regions – a one-year follow-up study

**DOI:** 10.1186/1471-2474-7-17

**Published:** 2006-02-23

**Authors:** Christina Gummesson, Sven-Olof Isacsson, Agneta H Isacsson, H Ingemar Andersson, John Ektor-Andersen, Per-Olof Östergren, Bertil Hanson

**Affiliations:** 1 Division of Physiotherapy, Lund University, Lund, Sweden; 2 Division of Social Medicine and Global Health, Lund University, Malmö Sweden; 3 Department of Health Sciences, Kristianstad University, Kristianstad, Sweden; 4 Department of Environmental Medicine, Lund University Hospital, Lund, Sweden; 5 Multidisciplinary Pain Clinic, Primary Care Region Skane, Malmö Sweden; 6 National Institute for Working Life, Stockholm, Sweden

## Abstract

**Background:**

The course of pain at a specific region such as the lower back has previously been shown as well as for generalized pain. However we have not found any report on the course of pain from various different specific regions. The aim of this investigation was to study the one-year transition of reported pain in different body locations.

**Methods:**

From a general population 14555 men and women, 46–68 years, responded to an extensive health questionnaire including the standardized Nordic questionnaire. The population represented 27% of the total population within the age group in Malmö, Sweden. At the one year follow-up 12607 responded to the questionnaire, yielding a response rate of 87%. The one year prevalence of long-lasting pain and the pattern of pain reporting from different regions were studied for men and women.

**Results:**

The one-year prevalence of long-lasting neck pain was 14% (95% CI 13–15) among men and 25% (95% CI 24–26) among women at baseline and 15% (95% CI 14–16) for the men and 23% (95% CI 22–24) for the women at follow-up. Of those reporting neck pain "all the time" at baseline, 48% of the men and 54% of the women also reported neck pain "all the time" at the one-year follow-up. At the follow-up neck pain was reported as present "often" by 43% of the men and 47% of the women who reported neck pain "often" at baseline. Similar transition pattern were found for neck, shoulders, elbow/wrist/hand and lower back symptoms, as well as consistent prevalence rates.

**Conclusion:**

The one-year transition pattern of reported pain was similar in different body regions and among men and women. Furthermore the prevalence rates of long-lasting pain in the population were consistent at baseline and the follow-up. The findings of similar transition patterns support the interpretation of long-lasting pain as a generalized phenomenon rather than attributed to specific exposure. This may have implications for future pain research.

## Background

Musculoskeletal pain is common in the adult population and one of the most common reasons to seek primary care[1]. The prevalence of long-lasting pain has been described according to specific locations [2-6] or as regional and widespread pain [7-9]. In cross-sectional general population studies it has been recognized that 67%-91% of the participants reporting long-lasting pain report more than one location [2,4-6]. However, specific locations and associated other pain locations have been sparsely investigated. In one general population study, 81% of those reporting long-lasting upper extremity pain also reported long-lasting neck, low back and/or lower extremity pain [4], indicating that long-lasting pain was often not localized to one region only. Pain in several regions has been recognized as a risk factor for more or long-lasting pain [9-12]. Long-lasting pain may be attributed to specific exposure or interpreted as a generalized phenomenon. Considering possibly similar risk factors for persistence of pain in different locations, similar transition of pain reporting in different body locations could be expected.

To provide further insight in pain transition pattern and persistence, different approaches have been used. Longitudinal general population studies of long-lasting pain have indicated a variation in symptoms between chronic, recurrent or transient when studying pain at one specific region [13,14] or generalized pain [9,13,15,16]. For example, at a one-year follow-up of participants reporting chronic widespread pain at baseline, 56% remained classified as having chronic widespread pain [15]. Similar results were found at a 3-year follow-up among responders reporting chronic widespread pain, where 45% were still classified as having chronic widespread pain [9]. Even in a study of chronic widespread pain with a 7-year follow-up, the finding was similar[16]. Thirty-four percent were still classified as having chronic widespread pain. However, widespread pain classification is dependent on the definition used. Mainly it is based on the American College of Rheumatology classification criteria which require pain to be present in at least two contra-lateral body quadrants and the axial skeleton. By this definition these results mainly imply that the number of reported painful regions fluctuate at least in some locations, however it does not give any information on the course of specific pain.

In studies focusing on a single location also a transition of pain reporting has been noted [13,14]. At a 2-year follow-up in a population reporting long-lasting neck/shoulder pain, 63% still reported neck/shoulder pain or generalized pain. Of those initially classified as having generalized pain, 85% remained classified having generalized pain [13]. A 5-year follow-up study describing the reporting and transition of low back pain [14] revealed a large fluctuation of the reporting of pain among the individuals. Of those reporting long-lasting/recurrent low back pain at baseline, 39% still reported long-lasting symptoms at the follow-up.

It seems well established that the prevalence of long-lasting pain is higher among women than men [3-5,7,13]. Though, we have not found any study on if transition patterns and persistence of pain are different or similar in different locations, among men and women.

The aim of this investigation was to study the one year transition of reported pain at different body locations.

## Methods

### Design

This epidemiological study (the Malmö Shoulder and Neck Study) was part of a large population study which took place 1992–1996. All inhabitants in Malmö born 1926–1945 were defined as the cohort, n = 53325 in the Malmö Diet and Cancer study. Recruitment was done by media campaign and personal invitations. The present study cohort consisted of all participants who were enrolled during February 1992 – December 1994 [17].

The focus on the information in the invitation was on the relation between diet and cancer. The questionnaires were designed to assess different aspects of health. The baseline questionnaire was administered at a medical visit and a follow-up was sent by mail one year later (median 12.6 months, inter quartiles 12.3–13.3). Two written reminders and one telephone reminder followed when needed. The questionnaires were immediately checked for missing data and completed by telephone interview by one investigator (AI), when necessary.

### Questionnaire

Musculoskeletal symptoms was assessed by the standardized Nordic questionnaire previously shown to be reliable and valid [18] inquiring about symptoms at different regions (neck, shoulders, lower back, and elbows/wrists/hands); "During the past twelve months, have you had any symptoms (pain, ache, or discomfort) in the neck? The five response options were adjusted from the original version, and were; "no, never", "yes, occasionally", "yes, sometimes", "yes, often" "yes, all the time".

Additional questions were included regarding sociodemographics and general health. For global health assessment an inquiry about self-reported health was used; "How do you feel right now, *physically and mentally*, considering your health and wellbeing". This item had seven response options, from "Feeling very bad" to "feeling very well". The complete questionnaire can be obtained from the authors upon request.

### Participants

The Malmö Shoulder Neck Study took place between February 1992 and December 1994 and included 14 555 participants 46–68 years old (27% of the total Malmö population within the age group). At the one-year follow-up 12607 responded to the questionnaire, yielding a response rate of 87%.

Assessment of possible selection bias was done by comparing sociodemographic data from the 1994 municipal statistics office. The variables compared to the total population of Malmö within the same age group were; gender, socioeconomic status (job title, task and position at work), and born abroad or in Sweden. The analysis showed that the Malmö Shoulder Neck Study cohort was representative of the total population in Malmö in the same age range, however those born abroad were underrepresented [19].

### Assessments

Prevalence rates for the one-year point prevalence were calculated as percentage of responses in each category. The responses indicating musculoskeletal symptoms "often" and "all the time" during the past twelve months were used to define long-lasting musculoskeletal pain, for the prevalence estimates.

The transition probabilities of musculoskeletal symptoms between the five response options from baseline to follow-up were assessed by percentage calculations for each category at each occasion.

For the assessment of self-reported global health, the item responses were dichotomized into poor health (response 1–3) and good health (response 4–7).

Test-retest reliability assessment was performed with a two week interval at the baseline study. A random sample of 232 participants was invited to complete the same questionnaires a second time. Two hundred eleven participated (90%). Kendall's Tau-B was used for the analysis.

### Ethics

The study was approved by Lund University Committee of ethics. All participants received written information. Approval for the data register was obtained from the Swedish Data Inspection Board.

## Results

The baseline and follow-up study were completed by 12 607 participants, mean age 57.2 (sd 6.0) years at baseline, 44% were men. Of the responders only participating in the baseline study 46 % were men, mean age of 56.6 (sd 6.0) years, Table 1.

The prevalence of reported long-lasting pain was similar at the two occasions. Thirty-four percent (95% CI 32–34) of the men reported long-lasting pain in any region at baseline and 32% (95% CI 31–34) at the follow up. Of the women, 46% (95% CI 45–47) reported long-lasting pain at baseline and 44 % (95% CI 43–45) at the follow up, Table 2.

**Figure 1:**
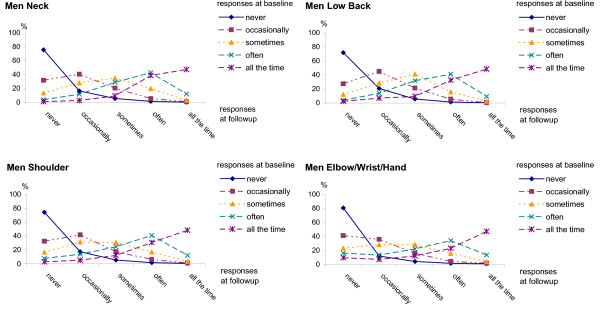
The course of reported symptoms among men; the reported symptoms at baseline and the transition in percentage at the one year follow-up. For instance, of those reporting neck pain "all the time" at baseline, 48% reported neck pain "all the time" also at the follow up.

**Figure 2:**
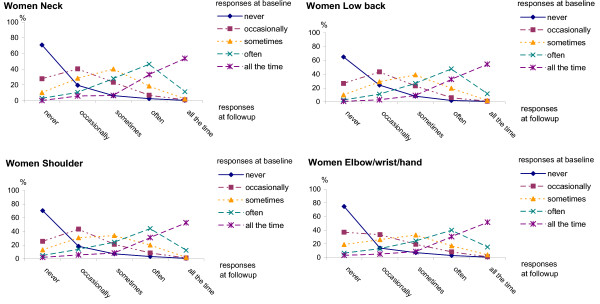
The course of reported symptoms among women; the reported symptoms at baseline and the transition in percentage at the one year follow-up.

The one-year transition between the symptom categories indicated individual changes in pain reporting, Figures 1 and 2. Of those reporting neck pain "all the time" at baseline, 48% of the men and 54% of the women reported neck pain "all the time" also at the follow up. Elbow/wrist/hand pain "all the time" was reported by 48% of the men and 52% of the women of those initially reported elbow/wrist/hand pain "all the time". At the follow-up neck pain was reported "often" by 43% of the men and 47% of the women reporting "often" at baseline. Of those reporting neck pain "sometimes" at baseline, 35% of the men and 40% of the women also reported "sometimes" at the follow-up. Similar transition pattern were found from all body regions.

Among the responders reporting long-lasting pain in the neck, shoulders, elbows/wrists/hands and lower back 34% of the women and 36% of the men reported poor health on the global assessment of self-rated health. An increase of reported poor health was found among those reporting more areas of symptoms compared to those reporting no symptoms or fewer regions. Among men, 10% (95% CI 8–12) of those with long-lasting pain in one region reported poor health. Of those reporting three regions with long-lasting pain, 25% (95%CI 20–31) also reported poor health, and 36% (95% CI 27–47) of those reporting pain from four region also reported poor health. Among women, 10% (95% CI 8–12) of those with long-lasting pain in one region also reported poor health. Of those reporting three regions, 25% (95% CI 20–31) also reported poor health and 34% (95% CI 28–40) of those reporting pain in four regions.

The Kendall's Tau-B was 0.80 for reporting of shoulder and neck symptoms, 0.76 for shoulder symptoms, 0.77 for neck symptoms and 0.70 for arm symptoms at the test-retest reliability assessment at the baseline study. For the item on self-rated health the Kendall's Tau-B was 0.72.

## Discussion

The aim of this investigation was to assess the pain reporting from several different locations, at baseline and at a one-year follow-up. The reported course of pain was similar in different body regions. For the studied regions, the transition patterns were similar for men and women. none was found to have more persistent pain than others or different pattern of symptom change.

In spite of similar prevalence rates at the two occasions, transition of symptom reporting had occurred. Transition of pain has in general population studies earlier been described for a specific body region [13,14,20] or by the use of widespread pain definition [9,15,16]. This study extends the previous findings to more detailed information on pattern of symptom transition from several specific body regions.

These findings add to previous literature as it, from a large general population, describes patterns of pain reporting from several regions, among men and women. In consistence with previous findings a higher prevalence of reported pain was found among women than men, for all regions [2-8,16,21-23]. Even though the prevalence of pain consistently was shown to be higher among women, the transition pattern appeared to be similar.

The exact prevalence rates will differ between different studies due to factors such as the use of different questionnaires and long-lasting pain definitions. In this study the symptoms were referred to as pain, ache or discomfort, possibly including more symptom-reporting than when the inquiry only reflects pain. However, in spite of study design differences, findings are in concordance regarding gender differences and which regions are most common, i.e. lower back, neck and shoulders [2,3,5,6]. In addition, some have described knee pain being among the most important pain regions [22,23], however that was not included in our study.

Poor general health has previously been reported to contribute to an increased risk of long-lasting pain, such as neck pain [11] or any long-lasting pain [6,11,24]. We studied how perceived global health was reported among those reporting musculoskeletal symptoms. In agreement with other studies, more responders reported poor health among those with multiple pain regions [9,25].

Our findings indicate that among persons experiencing pain even occasionally, the majority will during a one-year time still experience pain. Few move from pain "all the time" or "often" to "never" within one year. The current analysis focused on the reported musculoskeletal symptoms. For future studies the cohort of participants reporting increased pain is important to further investigate. In earlier occupational studies physical and psychosocial factors have been recognized as important for the reporting of discomfort from the neck and shoulder/arms [26]. Similarly it has been indicated that there is a strong interaction between physical and psychosocial risk factors and the onset of symptoms from the neck/shoulders/arms [17,27]. Also the influence of trauma as a risk factor has been assessed and described [28]. Because the transition patterns from the different body regions are similar in this general population sample, it would be important to further investigate risk factors for the different body regions simultaneously. The findings in the current study may reflect shared risk factors, such as depression. This study was not designed to explain the findings, rather highlight pain patterns among both men and women for different body region. When interpreting the results, it is important to consider that the results in this study may potentially be biased by the risk that if a person reports one symptom, they may be more likely to report several symptoms. Possibly also the perception of pain when rating several regions may be influenced by each other, so that changes in pain in one region affect the reporting in similar manner for other regions as well.

The common transition pattern for various body regions found in our study, may be difficult to explain by variation of exposure. The mechanisms of sensitisation and disinhibition of pain shown to be important in chronic pain status [29-31] could contribute to spreading of pain but also to a common variation of pain from different regions[32,33].

This study adds to the knowledge base among the community working on management of long-lasting pain. It confirms the previous well known fact that the prevalence of long-lasting pain is higher among women than men. In this large general population study the transition of pain ratings at the one-year follow-up indicated that the change is mainly to the next level, such as from "all the time" to "often" or no change at all, irrespective of location affected. The findings further highlights that this transition pattern of symptoms appears to be similar among men and women irrespective of location.

## Conclusion

The one-year transition pattern of reported pain was similar in different body regions and among men and women. Furthermore the one year prevalence rates of long-lasting pain in the population were consistent at baseline and the follow-up. The findings of similar transition patterns support the interpretation of long-lasting pain as a generalized phenomenon rather than attributed to specific exposure. This may have implications for future pain research.

## Competing interests

The author(s) declare that they have no competing interests.

## Authors' contributions

CG was responsible for manuscript preparation and parts of the statistical analysis. SOI was responsible for design and conduction of the study and was involved in early drafting and revision of the article. AHI was responsible for the study concept, assisted in the coordination of the study and data collection and was involved in drafting the article. HIA contributed to the revision the article. JEA contributed to conception and design and data collection of the follow up study and was involved in the revision. POÖ contributed to conception and design, data collection and was involved in early drafting and revision of the article. BH contributed to conception and design of the study and was involved in drafting the article. The MSNS group was responsible for the design and conduction of the study, and the data collection. All authors except BH (deceased) read and approved the final manuscript.

## Pre-publication history

The pre-publication history for this paper can be accessed here:



## Figures and Tables

**Table 1:** Age, gender and pain reporting for those responding to the baseline and the follow-up, and those only responding to the baseline survey.

	Baseline for responders to both surveys n = 12607	Baseline for those responding only to the baseline survey n = 1948
	
Age, years mean (sd)	57.2 (6)	56.5 (6)
Women/men, %	56/44	54/46
**Neck pain, %**		
Never	38	32
Occassionally *or *sometimes	42	42
Often *or *all the time	20	26
**Shoulder pain, %**		
Never	36	31
Occassionally *or *sometimes	43	42
Often *or *all the time	21	28
**Hand/elbow pain, %**		
Never	51	45
Occassionally *or *sometimes	33	32
Often *or *all the time	16	22
**Low back pain, %**		
Never	31	29
Occassionally *or *sometimes	51	44
Often *or *all the time	19	27

**Table 2:** Prevalence rates for long-lasting pain among men and women at baseline and one year follow-up.

	Baseline	One year follow-up
	Responders with Long-lasting pain**% (95%CI)**	Responders with Long-lasting pain **% (95%CI)**
	
**Men, n = 5593**		
**Total**	**33 (32–34)**	**32 (31–34**)
Neck	14 (13–15)	15 (14–16)
Shoulder	16 (15–17)	15 (14–16)
Elbow/wrist/hand	11 (10–12)	11 (11–12)
Low back	16 (16–17)	16 (15–17)
		
**Women, n = 7014**		
**Total**	**46 (45–47)**	**44 (43–45)**
Neck	25 (24–26)	23 (22–24)
Shoulder	25 (24–26)	24 (23–25)
Elbow/wrist/hand	20 (19–21)	20 (19–21)
Low back	20 (19–21)	21 (20–22)
